# Taunakitanga Takitini, Reframing Self-Management Support for All in Aotearoa New Zealand: Protocol for a Participatory Case Study Program of Research

**DOI:** 10.2196/89658

**Published:** 2026-04-08

**Authors:** Leigh Hale, Bernadette Huatau Jones, Ofa Dewes, Shirley Keown, Brigit Mirfin-Veitch, Amanda Wilkinson, Donna Keen, Pauline Norris, Eileen McKinlay, Meredith Perry, Kate Diesfeld, Henrietta Trip, Tristram Ingham, Nancy Longnecker, Roberta Francis Watene

**Affiliations:** 1 School of Physiotherapy University of Otago Dunedin, Otago New Zealand; 2 University of Otago Wellington Department of Medicine University of Otago Wellington New Zealand; 3 The Cause Collective Auckland New Zealand; 4 Te Hauora a Tūranganui a Kiwa Tūranga Health Gisborne New Zealand; 5 Donald Beasley Institute Dunedin New Zealand; 6 Va’a o Tautai – Centre for Pacific Health University of Otago Dunedin New Zealand; 7 University of Otago Wellington Centre for Interprofessional Education University of Otago Wellington New Zealand; 8 School of Community and Public Health Auckland University of Technology Auckland New Zealand; 9 Department of Nursing University of Otago Christchurch New Zealand; 10 Department of Botany University of Otago Dunedin New Zealand

**Keywords:** self-management, support self-management, Māori People, Pacific Island People, learning disability, disability, wellbeing

## Abstract

**Background:**

Increasingly, people are living with multimorbidity and long-term conditions or permanent impairments, which contribute substantially to health loss and additional health inequity. Critical to managing this health crisis is the appropriate support provided by health and social services. International research has informed the implementation of supported self-management knowledge into Aotearoa New Zealand. Meagre attention has been given to how New Zealand health care organizations can appropriately support people to self-manage their lifelong conditions within their own life contexts and cultures. Currently in New Zealand, those experiencing the greatest health inequities are Māori and Pacific peoples—Tāngata Whaikaha Māori (Māori living with disability) and people with learning (intellectual) disability who live with long-term health conditions or permanent impairments. This research aims to challenge and reframe supported self-management in New Zealand to enable our underserved populations and their whānau (families) to receive appropriate support to live well.

**Objective:**

We aim to reconceptualize supported self-management in New Zealand as a holistic approach to the provision of support and care within the complexities of the lived human and whānau experience. In total, three objectives will be considered across 3 key populations of people with long-term conditions or permanent impairments (Māori, Pacific Peoples, and those with learning disability) to (1) understand our community partners’ and their communities’ aspirations for living well and how best to support these desires, (2) develop innovative models of support by working alongside communities to enable supported self-management within their own context, and (3) implement models and evaluate outcomes.

**Methods:**

Embracing social construction of reality, this participatory case study program uses mixed methods and implementation evaluation design and is underpinned by Whānau Tuatahi (Māori) and Fonua (Tongan) research models and the Transformative Paradigm. Moreover, 3 case studies, 1 for each population group, will apply the same program objectives. Objectives 1 and 2 will be addressed with qualitative methodologies underpinned by relevant participatory designs. Objective 3 will use appropriate implementation frameworks.

**Results:**

Funded in October 2023, we have completed 2 years of this 5-year program grant. These first 2 years were focused on relationship building, ethical applications, research capability, and capacity building. Substantial, progressive consultation with the respective communities of each case study was undertaken.

**Conclusions:**

New knowledge generated across our program has the potential to inform New Zealand policy and practice about service delivery acceptable to the people to whom it matters (particularly Māori, Pacific Peoples, and people with a learning disability) and places emphasis on well-being promotion. This approach focuses on the inherent strengths and abilities of people, rather than the deficits or problems, acknowledging the wealth of expertise and experience people living with long-term conditions or permanent impairments bring, and builds relationships and partnerships between people and health care partners.

**International Registered Report Identifier (IRRID):**

PRR1-10.2196/89658

## Introduction

### Background

This paper describes the overarching protocol for a program of research aimed at building Aotearoa New Zealand–specific knowledge regarding supported “self”-management in the context of long-term health conditions or permanent disabilities. Intentionally, the research focuses on underserved populations, namely Māori and Pacific Peoples, Tāngata Whaikaha Māori (Māori with lived experience of disability) and people with a learning (intellectual) disability and their whānau (extended family) and how they can be optimally supported to live the best life possible. In this research, we seek to disrupt current thinking and understandings of supported self-management and reframe the concept specifically for the New Zealand context.

### Long-Term Conditions and Self-Management

Living with a long-term health condition or impairment (hereon collectively referred to as a long-term condition) impacts an individual personally (physically, mentally, emotionally, spiritually, and economically), their family (whānau), and the wider society and economy [1-3].

The impact of long-term conditions on the health of populations, and the associated costs of these, is an important determinant of public policy and public spending [4]. Underlying the interest in and recognition of the impact and cost of long-term conditions are neoliberal discourses [5], resulting in attempts to reduce the burden and attached societal costs arising from long-term conditions [6]. Thus, “defining and supporting patient self-management [has become] an important task of health services. Self-management [is now] a “policy relevant” construct, clearly within the remit of the health system and [therefore] one of the daily tasks of patients and health professionals in their encounters” [6]. Greaney and Flaherty [7], however, propose that

[A]an under resourced self-management agenda is arguably a means of covert rationing and morally unjust. When self-care is not performed by the individual, the health service does not always replace or supplement self-care activities … incomplete self-care becomes care left undone and renders the already vulnerable individual at risk.

Such discourses require critical scrutiny, given the demands that increasing numbers of people living with multimorbidity and long-term conditions will place on the New Zealand health system [7]. The prevalence and cost of long-term conditions are high [8,9], plus relatively higher for Māori and Pacific ethnic groups, older people, and those who face socioeconomic disadvantage [10].

Latest information on the Te Whatu Ora (Health New Zealand) website [11] refers to the New Zealand Ministry of Health’s recently developed suite of resources and tools for primary care teams, which provide information to build understanding of self-management support and how to embed this in primary care. This website then provides a link to resources located on the Healthify website (a New Zealand website run by a charitable trust providing evidence-based accessible health information and self-help resources) [12]. Here, self-management is defined as “taking charge of your own health and health-related decisions.” The reported key points about self-management and self-management support are (1) having enough knowledge and confidence to be actively involved in what happens to you, and how your health conditions are treated or managed; (2) understanding your conditions and the medicines you are taking and why. This involves working with your health care provider to set goals to achieve what you want for your own well-being and quality of life; and (3) it enables you to make informed choices about your health care and to live as well as you can with your health conditions. While this information suggests endorsement of the neoliberal approach to health previously referred to, it also infers that support is required for people to manage their health. Indeed, international research considers support provided to a person or persons to people to manage their health and wellness as a crucial component to enabling well-being [13].

### Supported Self-Management

As our genesis in this research, we refer to “supported self-management.” By this, we currently mean how health care teams and systems collaborate with an individual and their whānau to support their self-management endeavors, and the actions and informed decisions they take to care for and maintain their own or their loved one’s health and well-being [14]. This focus on the crucial support from others required by people in their efforts to live well contrasts with the more neoliberal rhetoric of “self-management,” a term engendering philosophical principles of self-responsibility, independence, autonomy, and self-determination, as generally endorsed by health systems [14-16]. Supported self-management applied with holistic support and care has the potential to enable flourishing mauri (the essential quality and vitality of a being) and reduce health inequity in New Zealand, particularly for those populations experiencing health disparities or living precarious lives [17-19].

The concept of self-management has had extensive global research, and numerous self-management programs have been developed [20-22]. The benefits of these programs are evidenced [13], but participation by those living in lower socioeconomic situations and indigenous cultures is low [23]. Barriers to participation include multistep referrals and uptake processes, environment and financial barriers, and discord between individual health beliefs and program content, which focus on symptom management and patient activation, and not on what matters to the person [24,25].

The term “self” implies individual responsibility and personal agency, a perception criticized for diminishing the need for social responsibility and collective action to support people with long-term conditions [15,26]. Furthermore, self-management is considered a Western construct necessitating adaptation for indigenous and ethnic minority groups [26]. Evidence now supports a capabilities-perspective approach to support self-management by developing a collaborative, supportive, and enabling relationship between the person with the long-term condition, their whānau and communities, their health care professionals or organizations, and the wider public health system [26-28]. Some good examples of such a holistic approach exist within primary health care in New Zealand [29-32]; however, supported self-management per se largely occurs within communities. Few primary care services or general practices have sufficient or prioritized resources to provide adequate holistic support or preventative habilitation to achieve and maintain well-being, resulting in intergenerational cycles of multimorbidity and fatalism within whānau [8].

From a rights-based lens, at the individual, family or whānau, and community level, what does this mean and what would be the optimal support required to enable a well life? Addressing this question lies at the core of our program of research.

### Community-Based Research

Answering our research question necessitates community engagement and involvement. Communities are important; they directly experience local challenges and develop local solutions to address health inequalities [29]. Health care in New Zealand in recent years has rapidly transformed and has strengthened the importance of community involvement. The New Zealand health reforms in 2022, driven by the health and disability inequities evidenced in the Waitangi Tribunal Report 2019 (WAI 2575) [33] and outlined in the Pae Ora (Healthy Futures) Bill in 2022 [34], presented new community-level health opportunities. An example was the creation of Te Aka Whai Ora, a government agency established in 2022 to lead and improve Māori health outcomes, responsible for commissioning Māori health services and monitoring the overall system for equity. Of note, it was disestablished in 2024. Other examples include the xrevised Ministry for Pacific Peoples, and Whaikaha – Ministry of Disabled People. Recently, the latter was significantly downsized in terms of responsibilities and resources [35]. While the COVID-19 pandemic in 2020-2022 exposed areas of health and social inequities and failings in accessibility of health care delivery, it also identified local community solutions to these challenges that informed future health service delivery. Māori, Pacific, and health and disability services directly experienced these health care changes and were forced to adjust their care delivery accordingly. These services thereby became experienced in identifying strength-based solutions. Furthermore, as these services are embedded within their communities, they have trusting relationships with and comprehensive knowledge of the people they serve.

Valuing the authority of the experiential knowledge of community-based Māori, Pacific, and health and disability services goes beyond community engagement and involvement to address research questions and calls for an approach underpinned by participatory action research [36]. Providing a research platform based on the principles of participatory action research was recognized as the most appropriate and effective approach to supporting community health services to engage with their communities. This type of methodology would enable these services to construct what they perceive is needed to best inform and support the health and well-being of their community members living with long-term conditions. From such an approach, innovative community-led solutions and advocacy can be developed [37]. These solutions can provide transformative health delivery representative of the priorities and values of health and social services and the communities they support.

### Upholding New Zealand’s Te Tiriti o Waitangi

Research undertaken in New Zealand must uphold Te Tiriti o Waitangi (New Zealand’s founding document) and is guided by Vision Mātauranga (a policy framework designed to unlock the innovation potential of Māori knowledge, resources, and people). Mātauranga Māori refers to Māori knowledge, ways of knowing and knowledge generation practices, and is a broad system that encompasses time, space, place, and discipline; it is thus a knowledge-generating system. This knowledge-generation should be done with care. As described by Mercier [37], it should

…address a problem of shared concern so that there can be equal input from contributors; cannot be solved by one knowledge system alone; have equitable outcomes; build capability and capacity; are underpinned by Treaty principles such as protection and partnership; have Māori in leadership roles; and crucially, are injected with human values of honesty, truth-seeking, kindness, generosity and humility.

Thus, in researching alongside community-based partners, mindful observation of the 5 principles of Te Tiriti o Waitangi (tino rangatiratanga [self-determination], equity, active protection, partnership, and options) and Te Mana Raraunga (Māori data sovereignty guidelines) [38] is critically important.

Our research program is a collaborative partnership between community partners and academic researchers who will provide the research platform for guidance, support, and mentorship as requested by our partners. Upholding rangatiratanga, the community partners have developed their own studies within this program of research and hold sovereignty, autonomy, control, and independence over their study’s aims, methodologies, processes, and data.

This program’s kaupapa (principles and ideas which act as a base or foundation for action) began with a clean slate agenda in 2019 at an in-person hui (meeting) of researchers and community partners who all held existing relationships and concurring philosophies about supporting self-management for health and well-being. The research evolved over regular and numerous in-person partner visits and Zoom (Zoom Communications, Inc) videoconferencing hui. Consequently, the core of the program is focused on 3 community partners—a Māori Health Provider, a Pacific community organization, and Ngā Tāngata Tuatahi, People First New Zealand (People First), supported by a national, independent, nonprofit organization which undertakes disability research and education.

During the early stages, we desired an appropriate program title to reflect its kaupapa. The program’s Māori researchers and advisors met to discuss. Included in this hui was a rangatahi (youth; Kāi Tahu, Ngāpuhi) raised in Kōhanga Reo and Kura Kaupapa Māori who suggested “Taunakitanga Takitini: reframing self-management support for all in Aotearoa” as a formal title, along with the whakatuaki (proverb) “E hara taku toa I te toa takitahi, ēngari he toa takitini” (“My successes are not mine alone, they are ours, the greatest successes we will have are from working together”). Later, the Kaumātua (elder) of the Māori health provider endorsed this title.

### Research Program’s Purpose

Our program endeavors to build mātauranga Māori and Pacific and disability knowledge and understanding of supported self-management for people living with long-term conditions within the context of New Zealand. As supported self-management is a human right [39], enhancing how the health system provides such support will, by respecting and enabling people or group-led solutions relevant to their context, contribute to reducing health inequities in New Zealand. For this, we must continue to challenge and evolve conventions of “self”-management, adopting holistic, culturally appropriate, and disability-centered approaches focused on people and whānau priorities and solutions.

### Research Program Aims

This program of research aims to reconceptualize “supported ‘self’-management” in New Zealand as a holistic and evolving approach to the provision of support and health care within the complexities of lived human and whānau experiences. The following specific objectives will be applied across three key populations of people living with long-term conditions, (1) Māori, (2) Pacific peoples, and (3) Tāngata Whaikaha Māori and people with learning (intellectual) disability to, first, understand the program’s community partners’ and their communities’ needs and aspirations for living well and how best to support people living with long-term conditions or disability. Second, develop innovative models of how health and social services can support and work alongside whānau and communities to enable supported “self”-management within their own context. And third, implement these models and evaluate outcomes.

More specific program research questions are presented in Textbox 1.

**Specific program research questions.**

- What are the aspirations and challenges to living well of Māori, Pacific peoples, Tāngata Whaikaha Māori and people with a learning (intellectual) disability living with long-term health conditions?
- How do these populations think health care services can best support them?
- What are the aspirations and challenges of the health, social, and advocacy organizations who currently support these populations?
- How can community health, kaiāwhina (support workers), and allied and nursing health care professionals best provide support to enable these aspirations?
- What can we learn from our community-based health care research partners who already have developed innovative holistic models of care for their service users, especially during these current transitions in health delivery?
- Together with our research health care and advocacy partners can we refine these models of health care or develop new ones to meet the aspirations of our targeted populations group?
- How can we measure the impacts of these refined or new models of health care?
- How beneficial are these refined or new models of health care?
- How can community providers use data sovereignty guidelines to safeguard the valuable data collected from their communities?
- And thus, what does effective “supported ‘self’-management” look like in New Zealand?

## Methods

### Overall Philosophical Approach

This research takes a sociocritical approach, applying a critical social construction worldview within the Transformative Paradigm [40]. The Transformative Paradigm combines diverse philosophical strands associated with feminism, critical theory, Indigenous and postcolonial theories, and disability and deafness rights theories [36]. Thus, this program of research is based on a participatory case study approach [41] that applies a mixed method design [42] and implementation evaluation [43].

### Our Methodological Approach

Given our philosophical approach, our methodological stance goes beyond cocreation or co-design with our 3 community partners to that akin to a participatory approach. Notwithstanding the lack of explicit definitions of cocreation or co-design in health care [44,45], these 2 methodologies imply an equal partnership between the communities concerned and the researchers. In our work, the 3 community partners have control and sovereignty of their own studies, including how their data are collected, analyzed, and used. The research team members’ roles are to support and facilitate the process; prepared to be reflective, flexible, accept uncertainties and pivot as required [36].

Thus, we are taking a participatory case study approach based on the social construction of reality [41]. Our program comprises 3 discrete case studies, representing our 3 targeted population groups, each sharing the same 3 program objectives. We will leverage the strengths of each case study to showcase what best practice and integrated support can look like as examples of New Zealand evidence-informed practice (“homegrown”) rather than imported initiatives and models that are not contextually relevant or understood. Our community partners’ current practices have been developed from working with their communities within their lived contextual experience. Our program of research is designed to generate a deeper understanding of what our partners do, what their communities aspire to, and what they want the health system to respond to. This information will enable exploration of existing gaps or successful strategies for the achievement of equitable health outcomes. This interconnecting and intersectional research will foster a New Zealand–focused community of practice, strengthening knowledge transfer between academics, partners, and government agencies, and with traditional health care delivery models (eg, general practice), acknowledging deficits of a “one size fits all” approach for good health outcomes.

While overall the research has as its foundation the Transformative Paradigm [40], each case study is underpinned by appropriate methodological approaches. For case study 1, this is Kaupapa Māori Research [46] and the holistic health model of Te Wheke (Māori) [47-49]. The Fonua health model [50] underpins case study 2. Case study 3 is based on the Transformative Paradigm. Using holistic models will enable us to explore the multifaceted and interactive components of mauri ora (healthy individual), whānau ora (healthy families), and wai ora (healthy environments; a collective Tongan concept is mo’ui lelei [51]) that determine or contribute to supporting self-management with the underlying intent of increasing social transformation.

### Ensuring our Research Is Culturally Safe

For our overall program of work, we are guided by the Whānau Tuatahi Research principles to ensure culturally safe research processes and effective outcomes for all involved [46]. This framework enables a practical adaptation and application of Westernized research methods within a Kaupapa Māori methodology and is formed by the following concepts—whakawhirinaki (trust), whakawhanaungatanga (building relationships), whakamana (empowerment), ngāwari (flexibility), utu (reciprocity), and hurihuringa (reflexivity).

### Our Conceptual Method

**Figure 1:**
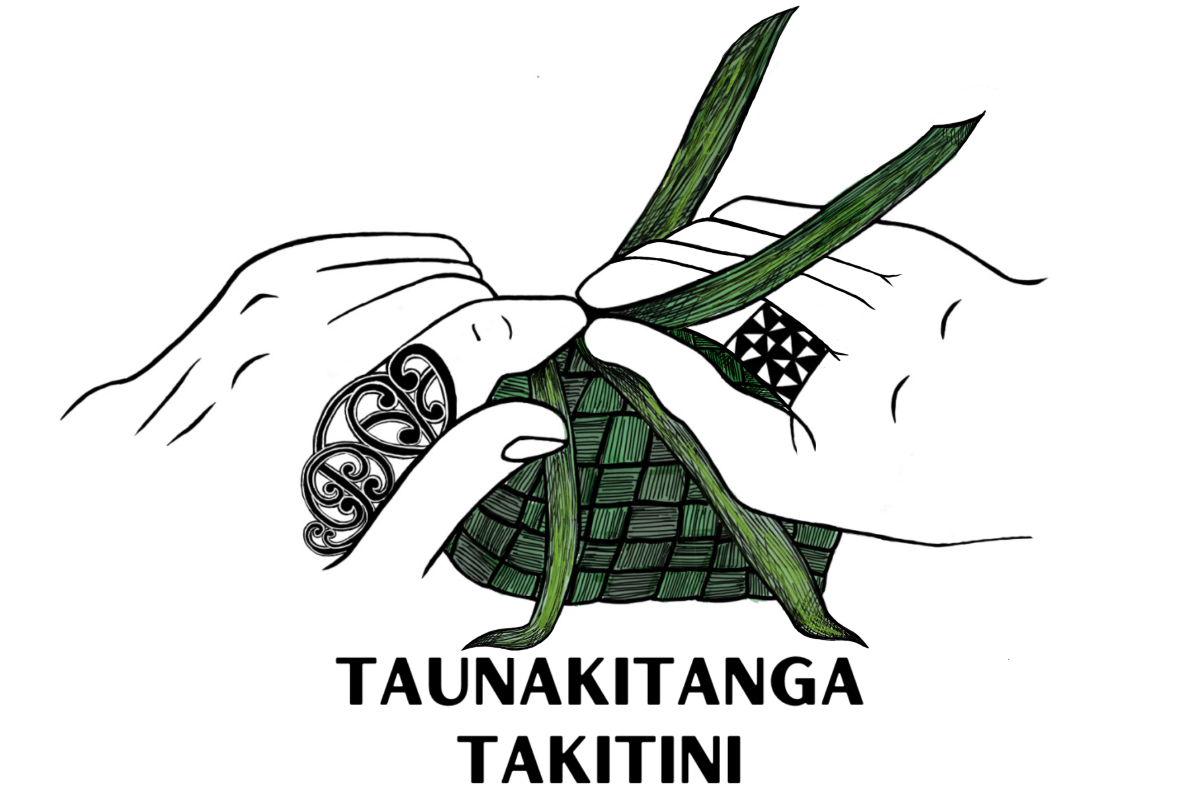
Taunakitanga Takitini (commissioned and created by Emily Fruean from Si’usega, Samoa).

These concepts are woven through each of our 3 studies, providing a supportive whariki (mat). Figure 1 depicts an unfinished woven mat, a traditional art form creating functional implements for both Māori and Pacific Peoples. While most of the mat is woven and supportive, acknowledging the foundational strengths and knowledge that currently exist in our community partner organizations, the unfinished sections represent gaps in knowledge for which strands still need to be woven to provide more synergistic and supportive linkages to enhance resilience for our 3 population groups.

Our research program endeavors to progress toward completion of this mat by tightening the intersectional weave. While traditionally used as a ground mat, woven mats are also used as sails in traditional boats. The turbulent seas of the transforming health services and the pandemic challenges require a strongly woven mat that ensures we sail and land together safely, especially for oppressed and underserved populations with long-term conditions.

### Case Study 1: Kaupapa Māori Research

Kaupapa Māori Research methodology underpins much of this program of research. It is a transformative methodology that is conducted by, with, and for Māori to benefit the Indigenous Māori people of Aotearoa New Zealand [52]. It is grounded in Māori philosophical principles and seeks to address the negative impact of Western research practices that have historically misrepresented and exploited Indigenous peoples [53]. Kaupapa Māori Research seeks to give voice to Indigenous Māori who have been historically silenced, particularly those most negatively impacted, such as disabled Māori and women’s subgroups [54]. This methodology promotes Māori self-determination over the entire research process with the focus on privileging the voice of Māori and supporting mana-enhancing outcomes [52].

A range of Māori models of health will be drawn on throughout this program, including Te Wheke. The Te Wheke model is a holistic Māori health and well-being framework that uses the wheke (octopus) as its central metaphor [47]. The head of the octopus represents the whānau, emphasizing its central role in supporting health and well-being. The eyes of the octopus symbolize waiora (total well-being for both the individual and the whānau). Each of the 8 tentacles represents a specific and interconnected element of health—wairuatanga (spirituality), hinengaro (the mind), taha tinana (physical), whanaungatanga (extended family), mauri (the life force), mana ake (unique identity), hā a koro ma, a kui ma (the breath of life from ancestors), and whatumanawa (the expression of emotion). The interwoven nature of the tentacles signifies that these dimensions are deeply connected, acknowledging that health is an integrated concept in Te Ao Māori [the Māori world]. This holistic approach contrasts with Western biomedical models by prioritizing the link between the physical, the mind, spirit, whānau, and the wider world [47].

Interpretation and analysis of data generated from this program will also be cognizant of holistic Māori models of health, well-being, and disability that privilege the voices of participants in this program of research. For example, Te Pae Mahutonga, a Te Ao Māori health promotion framework, was used to interrogate health and disability outcomes for disabled Māori and thereby inform equity in health policy and services [55].

Māori models of health and well-being are highly relevant for this program of research because they are collective in nature and prioritize the interconnectedness of individuals within their social and environmental contexts. These models encompass critical cultural practices and concepts, including tikanga (protocols), te reo Māori (the Māori language), mātauranga (knowledge), whakapapa (genealogy), and social connectedness. They are flexible in their application and can also be adapted to encompass and respect other cultures and collective communities, such as disabled groups and Pacific Peoples. While existing models can be a useful guide, this research aims to explore the extent to which these models meet the needs and aspirations of the targeted populations and whether we need to refine or develop new models to deliver maximum benefits to our health care delivery.

### Case Study 2: The Fonua Health Model

The Pacific component of our research program is characterized by (1) leadership-driven (because of the mana-enhancing social structures in Pacific families and communities), (2) systematic engagement of relevant stakeholders (because of the need to involve our stakeholders in the change process to achieve wide coverage and sustainability), (3) transferable processes (to other populations living in persistent disadvantage), and (4) culturally mediated and community-delivered (because of the pivotal role that Pacific communities have on cultural norms).

Our cultural approach is to ensure the voices of Pacific communities, especially those living with long-term conditions and the services that currently support them, are listened to, understood, respected, and assisted as to how best to reframe supported self-management from their perspective. Active listening will build confidence, courage, and trust between participants, the services, and researchers, resulting in mutual understanding and meaningful engagement and communication. Moreover, it will encourage and activate creative thinking about new ways of providing supported self-management that will enable people living with long-term conditions to live their lives in the best possible way. Bilingual talanoa (a process of inclusive, participatory, and transparent dialogue) will align with Pacific and New Zealand Health Research Guidelines [56] on communal relationships, cultural sensitivity and reciprocity, and the Fonua health model.

The Fonua model is a Tongan framework comprising 5 dimensions of life that are interwoven like a mat and interdependent and complementary of each other [49]. The 5 dimensions are Laumalie (spiritual), ‘Atamai (mental), Sino (physical), Kāinga (collective or community), and ‘Atakai (environment). Within these 5 dimensions are five structural levels that are (1) Taautaha (individual), (2) Famili or Kāinga (family), (3) Kolo (local community or village), (4) Fonua (nation), and (5) Mamani (global society) [50]. Thus, our laakanga [weaving] phases of research will be guided by the following processes.

1. Kumi Fonua (consultation): We will establish connections with our community leaders and key stakeholders face-to-face. Talanoa will comprise information to guide the cocreation of a quality framework for interaction during this activity. More specifically, we will seek to understand what supported self-management might look like for people living with long-term conditions, with a view to then creating a model of health care that will best develop, deliver, maintain, and support a community-wide generic program of supported self-management.

2. Langa Fonua (data collection): We will ascertain what data should be collected and analyzed. Our design will be augmented by a community-based social movement framework that will include people living with long-term conditions and their primary caregivers who live with them.

3. Tauhi Fonua (data analysis): Data will be analyzed for common themes, discussed, and refined. Reflections on the knowledge generated will enable deeper understanding. Furthermore, the notion of sustainability, along with the roles of support and community workers, and health care professionals in this support, will be explored and made explicit. The outcome will be a refined or new model of supported self-management, with decisions made on the evaluation methods for the new model. Final analysis will include recommendations on how the results may be delivered for implementation and evaluation.

4. Tufunga Fonua (dissemination or reformation): Final analyses will include recommendations on how the results and the new model of supported self-management may be delivered for implementation and evaluation. This will include feedback to stakeholders, presentations at community fonos (meetings) and conferences, publication via local media networks (print and radio), and scientific journals.

### Case Study 3: The Transformative Paradigm

Case study 3 focuses on people with a learning (intellectual) disability. The relationally based, inclusive approach of the transformative paradigm was considered appropriate as researchers and the community (participants) collaborate on an equal basis. The transformative paradigm has a social justice orientation and advocacy for marginalized voices, and it aims to achieve social change through research [40]. This framework purposively includes the “hidden” or seldom-heard voices, and contests aspects of power and privilege to promote social justice. Crucial to this approach is the building of relationships of trust and the valuing of lived expertise, not only from the standpoint of lived experience, but of acknowledging leadership, self-determination, and appropriate remuneration, and the reshaping of the power imbalances [40,57,58].

The researcher in the Transformative Paradigm is a disruptor for social justice who challenges inequalities while assuming shared responsibility and acting with humility [59]. The central tenet of the Transformative Paradigm is that power must be attended to at each stage of the research process; Mertens [40] challenges researchers to ethically examine who they are and who they are in relation to the community in which they are working. Relationship-based research practice underpins the Transformative Paradigm, ensuring authentic and caring relationships between the researchers and those with lived-expertise in participatory-based research [60,61]. The Transformative Paradigm provides a strong theoretical basis for the program’s study involving people with learning disabilities.

### Individual Backgrounds to the 3 Case Studies

#### Overview

The background to each case study is described below. The detailed methodologies and methods each study will take will be dependent on their initial and ongoing consultations with their communities and will evolve with time, and thus, these details will be presented in later disseminations as determined by each community partner involved.

#### Case Study 1: Hikoi Kite Oranga Tangata Katoa – Pakeke Matihiko

Turanga Health, a long-established and trusted iwi health provider in Tairāwhiti [62], will undertake the Māori-specific case study.

We were cognizant that supported self-management needs to be flexible and responsive to the sociocultural contexts of different people and cultures. Within Te Ao Māori, the concept of whānau encapsulates the whānau whānui (the broader or extended family and friends). The premise of supported self-management’s focus on “self” appears incongruent in a Kaupapa Māori context [63]; and maybe better defined as “whānau-support.” However, in current times, does this assumption of whānau-support hold true? Do time-poverty, financial stresses, urbanization, and the need for more whānau members to be in employment reduce the level of support that whānau experience and provide? Turanga Health considered that a better understanding of these aspects for different people would inform them (and other providers) as to how they could better support whānau wellness and overall well-being.

Working toward hauora (well-being) is a journey, and often a long journey for many people living with a long-term condition. Some whānau have a large and supportive whānau network able to engage, awhi (support), and tautoko (endorse) their whānau on their journey. Some whānau live without any support, while others have family members who, for various reasons, may not be able to provide the necessary or desired assistance (eg, due to work commitments, lack of space, or geographical distance). The uniqueness of these journeys was amplified during the COVID-19 pandemic. This study provides an opportunity for mana Motuhake (whānau determining their “what and how” for their hauora journey), rather than a “Westernized” model of supported self-management imposed on whānau.

The COVID-19 experience and the recent health reforms have caused reflection on current models of health care and how these could be modified based on recent experiences and learnings, and the future directions of health care. Turanga Health’s care is underpinned by the fundamental belief that understanding and being connected to the past are important for both the present and the future, as reflected in the whakatauki (proverb)—“*Turanga mua, Turanga tika, Turanga muri, Turanga he*.” (*Those who move forward and accept the challenge will bear the fruits of success. Those who do not advance will not develop*.)

#### Case Study 2: Lūsia Ki Taulanga

Lusia ki taulanga is a Tongan proverb referring to seafarers’ struggle through stormy weather to reach land, striving to achieve despite life’s challenges.

A Pacific case study will be undertaken in collaboration with The Cause Collective (TCC). TCC is a Pacific community organization in South Auckland “that aims to create the conditions for communities to thrive from this generation to the next.” TCC is interested in the overall outcomes of Pacific Peoples across Aotearoa New Zealand, underserved populations, and South Auckland communities [64]. With a vision that “This Generation will Win the Next Generation,” TCC is poised to accelerate the pace of change by co-designing, testing, and prototyping scalable breakthrough solutions to complex problems and achieve its goal of “healthy and thriving families and communities.” To get to the heart of pervasive and complex problems, TCC uses social innovation tools, critical thinking, and cultural perspectives.

TCC is also the only Pacific-led primary health care organization in New Zealand. Established in 2010, TCC is passionate about improving health equity and well-being for Pacific Peoples and other persistently disadvantaged populations. TCC provides tailored tools, guidance, and support to their network of 46 General Practices with funding, contract management, clinical and administrative tasks, education, business and clinical modeling, and service delivery and reporting so that they can achieve equitable outcomes for their >140,000 enrolled patients and local communities.

Under the umbrella of TCC, Tātou Collective was appointed by the government in July 2025 as the only Whānau Ora commissioning agency for Pacific Peoples in Aotearoa. Whānau Ora is a culturally based and whānau-centered approach to well-being focused on whānau (family group) as a whole, as the decision-makers who determine their goals and aspirations [65]. As an independent Pacific agency, Tātou Collective is focused on getting support to those with the greatest need, guided by transparency, integrity, and the best available evidence.

Aligning with Pacific and New Zealand Health Research Guidelines and bilingual Talanoa [56], the Tongan Fonua [50] research approach will be implemented. Fonua is a cyclic, dynamic model of interdependent relationships (va) between humanity and its ecology for the ultimate purpose of harmony in health and well-being. Fonua’s values are Fe’ofa’ofani (love), Fetokoni’aki (reciprocity), Faka’apa’apa aki (respect), and Fakapotopoto (wisdom and prudence) [50,56]. This study will draw on the strengths of Pacific Peoples, in which capabilities and knowledge abound. The Pacific capabilities lens suggests that, despite lower educational levels overall, Pacific Peoples, as a group, hold a wealth of knowledge, values, and experiences [49]. Gifting this to people living with a long-term condition is a resource and a means of empowering pathways of future-thinking and transformational impact.

#### Case Study 3: Supported Decision-Making to Increase and Enhance Supported Self-Management With People With Learning (Intellectual) Disability

Our community partners in this case study are an independent disability research organization, the Donald Beasley Institute [66] and Ngā Tāngata Tuatahi – People First New Zealand (People First) [67]. People First is the only learning disability disabled person’s organization (DPO) in New Zealand. DPOs have special status in New Zealand as organizations and are defined as a DPO if they are exclusively governed by and for the group of disabled people they represent. People First endorsed both the wider program, and case study 3, which is specifically concerned with and involves people with a learning disability. They are centrally involved in the research, including nominating People First members for a research advisory group, providing direction and advice throughout, and engaging in co-design and coproduction activities at all phases of the study, including the development, delivery, and implementation of the intervention.

People with learning (intellectual) disabilities comprise about 2% of New Zealand’s population [68], and experience poor health outcomes [68] mostly attributable to inequities in access to health information, services, and support [70]. People with learning disabilities are frequently perceived to lack the capacity to make decisions, including health-related decisions [71]. International research has also confirmed that people with learning disabilities experience inequities in accessing supported self-management programs [72]. This inequity is seen as a direct consequence of disability and health professionals perceiving people with learning disabilities as not having the capacity to receive or apply health information, or to make competent health-related decisions.

The presumed and conditioned incapacity experienced by people with learning disabilities is a significant barrier to supported self-management initiatives. Despite this, research has shown that people with learning disabilities can intentionally engage in supported self-management initiatives with successful outcomes [73-75]. Disability and health care professionals could facilitate supported self-management endeavors with individuals and their whānau (or other close supporters) [76]. A potential strategy to facilitate supported self-management is supported decision-making (SDM). SDM is a framework whereby disabled people with cognitive impairment are supported to make decisions that exercise their legal capacity and promote their rights, will, and preferences [77-80]. In prioritizing individual’s will and preference, SDM ensures disabled people remain central to decisions about them.

SDM challenges a “best interests” approach whereby decisions, including health decisions, are made by others according to their perceptions of what is “best” for the disabled person. There is an obvious synergy between supported self-management and SDM. Recent research in the learning disability and supported self-management arena points both directly [79] and indirectly [80] to the potential of SDM, but it is yet to be comprehensively studied. This learning disability–specific case study is designed to explore the potential of SDM to increase and enhance access to supported self-management with people with learning disabilities, inclusive of tāngata whaikaha with learning disabilities. Ensuring supported self-management is available to all as a human right meets aspirations of Te Tiriti o Waitangi and the United Nations Convention on the Rights of Persons with Disabilities.

### Overall Methods and Designs

#### Overview

This is a participatory case study research program [41] with an embedded QUAL quant mixed method [42] and implementation evaluation design [43]. Our 3 discrete case studies share the same 3 program objectives and are broadly structured around a similar phased design. This enacts our metaphor of the sail as a weave in process, but recognizing the need to first consider our threads and materials (objectives 1 and 2), then collaboratively weave together those threads, and testing them for strength and resilience (objective 3), with the hope that they will see us through to the next step of our journey.

This weaving metaphor operates within our guiding Whānau Tuatahi Research principles that ensure culturally safe research processes and effective outcomes for all involved [46]. This framework enables a practical adaptation and application of Westernized research methods within a Kaupapa Māori methodology and, as noted earlier, is formed by the following concepts: whakawhirinaki (trust), whakawhanaungatanga (building relationships), whakamana (empowerment), ngāwari (flexibility), utu (reciprocity), and hurihuringa (reflexivity). These concepts underpin our interactions with one another in our participatory approach to this research. These concepts are strong threads that underpin our 3 case studies and who we support through the mechanisms of hui, newsletters, strong communication, open leadership, advisory groups, and developing potential and capacity.

To address objectives 1 and 2, in each case study, the community partner will consult and explore their service users’ and communities’ perceptions, needs, aspirations, and desired health outcomes using qualitative methodological approaches, appropriate to the community in question. Based on these findings, each case study will then refine or develop health care models that can more appropriately support the aspirations of their people living with long-term conditions to live well.

To address objective 3, each case study will implement its developed or refined model and evaluate outcomes over 1 year. Given the research’s kaupapa (guiding principles), we were unable to predetermine which research methodologies and methods would work best in each study to achieve this third objective. While all 3 case studies will broadly follow the implementation process described below, each study will have a nuanced approach. Based on guidance from extensive community consultation to date, the following description for addressing objective 3 provides a suggested method, acknowledging that flexibility and agility will be required to be responsive to each case study’s communities’ directions.

#### Design

We followed the 12-step process proposed by Hudon et al [41] for conducting case studies with a participatory case study approach. To enact step 6, choosing a theoretical framework to guide data collection and analysis, we chose implementation science methodology as (1) the health environment, systems, and delivery are changing too rapidly to justify lengthy randomized controlled trials and (2) our previous experiences of randomized controlled trials in collectivism-based populations has identified the aversion and mistrust these communities have in such a positivist, individual-focused scientific enquiry [81].

As a starting point, we chose the RE-AIM (reach, effectiveness, adoption, implementation, and maintenance) model as a framework to evaluate site community impact [43,82]. The RE-AIM model is a robust, frequently used starting point for evaluation [83]. The RE-AIM dimensions ensure a holistic evaluation of health care interventions based on both qualitative and quantitative outcomes. Ways to evaluate these outcomes will be modified depending on each community’s wishes and requirements. Using a quasi-experimental design with each case study as a control group is not feasible given the diverse nature of the partners’ services.

#### Participants and Recruitment

While these details will be dictated by the desires of our community partners (finalized while addressing objectives 1 and 2), each implementation study will most likely include up to 20 participants who experience long-term impairments or conditions and their whānau. This sample size was dictated by (1) previous studies using a participatory case study approach with people with long-term complex health needs, which had sample sizes ranging from 10 [84] to 30 [85] participants, and (2) the size and capacity of our 3 case study community partners. Appropriate ethical procedures will be followed to invite participation and gain informed consent.

#### Intervention and Outcomes

The refined or new model of health care will be implemented within each community. As the program of research aims to reconceptualize “supported ‘self’-management” in New Zealand as a holistic approach to the provision of support and care to enable people with long-term conditions to live well, the primary outcomes of well-being and vitality will be evaluated using qualitative longitudinal research [86,87] with qualitative data collected longitudinally every 3 months over a 1-year period. We will also simultaneously collect quantitative data; the quantitative outcome measures to be used will be identified based on community consultation outcomes. Thus, for each case study, 80 individual semistructured interviews are likely to be undertaken; however, this is participatory research and the actual methods of data collection, as detailed in step 8 of the approach proposed by Hudon et al [41], will be finalized by our community partners during their consultation stages. The quantitative data collected via questionnaires will be contextually applied, as the small sample sizes will likely preclude comparative analysis.

For example, case study 1 (*Hikoi kite oranga tangata katoa – Pakeke matihiko*) may use qualitative data gathered via audio-recorded focus groups and semistructured interviews, photovoice, and pūrākau (storytelling), underpinned by a Kaupapa Māori Research paradigm and quantitative questionnaires, such as Hua Oranga (A Māori Measure of Mental Health Outcome) and Whānau Capacities [88].

Case study 2 (*Lūsia ki taulanga*) may use qualitative data gathered by the Pacific oral tradition of Talanoa, likely to be via audio-recorded focus groups and semistructured interviews, photovoice, and digital storytelling workshops [89]. Quantitative data may be collected via the Pacific Identity and Wellbeing Scale [90-92]. A culturally appropriate self-report measure assessing 5 factors (perceived familial well-being, perceived societal well-being, pacific connectedness and belonging, religious centrality and embeddedness, and group membership evaluation) of Pacific identity and well-being. Face, content, and construct validity are established for this measure, and exploratory and confirmatory factor analyses support the model.

For both case studies 1 and 2, other questionnaires that may be used include the World Health Organization-Five Well-Being Index [93,94]. This is a short self-reported measure of current mental well-being for use in primary care settings that has robust psychometric properties across a range of countries. And the Assessment of Life Habits (version 4.0) assesses life habits from daily activities to social participation across 12 domains [95,96].

Within the frameworks of inclusive disability and participatory research, the case study 3 team will evaluate the uptake and impact of SDM as a tool to support self-management of long-term health conditions. Specifically, new disability-led education and training for people with a learning disability and for supporters, including family and whānau, support workers, and health professionals, will be explored using RE-AIM principles to evaluate the potential of SDM in advancing supported self-management approaches that recognize and respond to people with a learning disability [97].

#### Data Analysis

Demographic and questionnaire data will be analyzed with descriptive statistics (means, medians, ranges, and frequencies). Differences in living arrangements, migration status, contact with home nations, economic status, and degree of the long-term condition that shape experiences of supported self-management may be explored. Audio-recordings will be transcribed verbatim. The qualitative data analysis will be undertaken by 2 culturally appropriate, trained research team members (Māori, Pacific, and learning disability) for each case study, following the reflexive thematic analysis approach described by Braun and Clarke [98,99]. Although this is a “Western” approach to analysis, notwithstanding reservations expressed by some Māori and Pacific researchers [100], many ingenious studies have used it, considering that its processes are appropriate and complementary [101-103]. Similarly, it is used frequently in research into learning disabilities [104,105]. To gain a sense of the overall themes, the analysis will begin with repeated listening of the audio-recordings and multiple readings of the transcripts. Important statements will be highlighted and provided with a preliminary naming code. With ongoing discussion and reflection by the research team, these initial codes will be collapsed and categorized to create a final coding scheme, which will then be applied across all transcripts within each case study. Subsequent case study research team reflective discussions will collate these codes into themes. Multiple team discussions, ongoing presentations, and discussions with the study advisory groups and community members will enhance the reflexivity, trustworthiness, and robustness of the analysis. Analysis and reporting will be in accordance with the Big Q Qualitative Reporting Guidelines. These guidelines articulate a values-based, rather than consensus-based, framework for reporting and evaluating qualitative research. They were developed to support methodological congruence and reflexively open evaluation and reporting of Big Q Qualitative research. Big Q Qualitative research includes both the techniques and values of qualitative research and thus rejects objectivist and positivist assumptions, values, and norms [106].

Each study may choose other uniquely diverse methods of evaluating outcomes appropriate to their context and what their communities considered best reflected living well for them, for example stories, dance, artwork, kapa haka (living Māori performance art form encompassing traditional songs, dances, and actions that express language, culture, and heritage), waiata (Māori song), theatre, music, or songs.

Hui and fono (meetings) will be held with participants and research teams to moderate analyzes and interpret the impact of implementation and evaluation, and kōrero or talanoa (discuss) about values, successes, challenges, and share improvement recommendations.

### Ethical Considerations

The broad program proposal has been registered and approved by the University of Otago Human Ethics Committee (HE24/002). Ethics for each phase of the research will be specifically applied for and each phase will be undertaken in accordance with the ethical standards of the responsible ethics committee and with the WMA Declaration of Helsinki. To date, this has included approval for the co-design research pertaining to objectives 1 and 2: University of Otago Human Ethics Committee (Health), category A: “Taunakitanga Takitini: reframing ‘self’-management support for all in Aotearoa” (H24/002); “Lūsia kiTaulanga: A Pacific study in ‘supported’ self-management” (H25/0403); and “Exploring Turanga Health values and ‘capabilities’ for evaluating program outcomes” (H24/0260). The research entitled “Reimagining supported self-management of health and wellbeing with people with learning disability in Aotearoa New Zealand” was approved by the Central Health and Disability Ethics Committee (New Zealand Ministry of Health; 2025 FULL 22164).

Ethical approval from the University of Otago School of Physiotherapy Ethics Committee (“The ‘Taunakitanga Takitini’ research team members’ reflections and engagements about self-management: SoP/EC/2024/09”) has also been approved to allow us to capture how our broad team of research members and partners change and grow into their aspirations and understandings of what reframing of self-management support could mean.

In all above ethically approved projects, informed written consent will be obtained from participants. Potential participants are informed of their right to refuse to participate in the research or to withdraw consent to participate at any time without reprisal. They are also informed that every precaution will be taken to protect their privacy and the confidentiality of their personal information. Participants will be reimbursed in recognition of expenses incurred by participating in the study, usually by way of a voucher.

In the research involving adults with a learning disability, as legally required in New Zealand, participants will be consented in a range of ways depending on their level of understanding, including (1) providing independent signed consent, (2) their legal guardian will provide consent, or (3) a consent statement will be provided by a significant other in consultation with the participant. We will provide a plain English study information sheet and consent forms approved by the Ministry of Health’s Health and Disability Ethics Committee. Every effort will be made to secure freely given informed consent, which may be verbal or written as appropriate, that participants have actively provided, and we will ensure that they have the time and opportunity to access support in their decision-making, for example, by discussing their choice with a trusted adult or relative.

## Results

This 5-year program grant was funded in October 2023. The first 2 years have been devoted to relationship building, ethical applications, research capability and capacity building, and data collection and consultation with the respective communities of each case study. We have completed our first program objective and partially completed our second objective.

## Discussion

### Principal Findings

Our program of research aims to build New Zealand–specific knowledge about supported “self”-management to enable those who experience the greatest health inequities, that is, underserved populations with long-term conditions, such as Māori and Pacific Peoples, Tāngata Whaikaha Māori and people with a learning (intellectual) disability and their whānau to be supported to live the best possible life. We anticipate that our 3 case studies will develop innovative approaches to support people living with long-term health conditions or permanent disabilities within their communities to live well; approaches that their community members perceive to be acceptable and beneficial.

We aim to disrupt current understandings of supported “self”-management and reframe the concept specifically for New Zealand to enable the vision of Pae ora (good health and well-being). Increasingly, people are living with multimorbidity, long-term conditions, or permanent impairment, which contributes substantially to health loss and additional health inequity [107]. Health and social services providing appropriate support is critical to managing this health crisis [108].

To date, international research has largely informed self-management knowledge and implementation in New Zealand, with little attention given to how New Zealand health care organizations and social services appropriately support people to self-manage their long-term conditions within their own life contexts. Reframing supported “self”-management with the New Zealand world view to include perspectives of Te Ao Māori, Pacific Peoples, and people with learning disabilities is crucial if we truly wish to enable the vision of the Pae ora (healthy futures or healthy life) Act. The current transformational health context, driven by the 2575 Waitangi Tribunal Report and the Pae Ora Act, the COVID-19 pandemic and subsequent economic crises, and the Royal Commission into abuse and neglect of children, young people and adults in the care of the State and faith-based institutions in New Zealand between 1950 and 1999, along with changing governmental agendas, presents a unique opportunity to reimagine health care access for people most at risk.

### Dissemination Plan

A clear pathway of planned activities will generate outputs and outcomes for research end users and knowledge transfer both within our program and wider. Reports and presentations will be iteratively constructed within each case study to ensure the voices and paradigms of the participants remain clear throughout. Rigorous processes will uphold community partners’ sovereignty and data ownership. Dissemination processes of hui, fono, meetings, and presentations will verify and shape findings. Appropriate findings will be gifted by the community partners and their communities to participants, key stakeholders, policy advisors, the funder and other health service providers.

### Future Research

The dissemination processes are expected to increase the impact of this research and ensure reciprocity through bidirectional learning, bicultural communication, and bidirectional feedback. Such exchanges provide a pathway for impactful transformational health knowledge transfer, whereby mindsets of health and disability providers and policymakers, organizations, professionals, and individuals or whānau are empowered for future health and well-being thinking. Future research should focus on the evaluation of these expected impacts on the perceptions, processes, and policies of health and disability providers and organizations and health professionals. Additionally, research to establish the social return on investment in delivering the innovative approaches to supporting people living with long-term health conditions or permanent disabilities is warranted, and this, in turn, will inform health policies.

### Strengths and Limitations

The strength of this research lies in its equity-focused methodology. It explicitly targets health equity by addressing issues identified by and for communities experiencing ongoing health and disability inequities; empowering them to drive the research kaupapa (agenda), building research capacity and capability, and developing health care models contextually relevant for New Zealand that have the potential to positively impact the health and well-being of disadvantaged populations. Impacts will be achieved by working in close partnership with health and disability providers and advocacy services, and by targeted dissemination of findings back to communities, providers, and policymakers.

Furthermore, our work program has a strength-based stance. Evidence from “Western” studies now supports a capabilities approach to supported self-management (developing collaborative, supportive, and enabling relationships between individuals with long-term conditions and health care partners) for improved health outcomes [13,19,25,26]. Reframing this approach from the perspectives of Tāngata Whenua, New Zealand’s Indigenous Māori population, and Pacific Peoples, collective approaches and those with learning disabilities have huge potential to foster self-actualization, maintaining and enhancing health and well-being for these populations to live richer, fuller lives; populations frequently underserved in the current delivery of health and disability services. Importantly, drawing on the wisdom of people who emanate from collective and supportive societies with deeply held values and binding genealogical, land, history and spiritual ties [109] will reframe supported self-management in ways that will be beneficial to enhance health and well-being and improve equitable outcomes for all living in New Zealand.

New knowledge generated across our program has the potential to inform policy and practice about service delivery acceptable to the people to whom it matters (particularly Māori, Pacific Peoples, and those with learning disability) and places more emphasis on promotion of well-being. This approach focuses on the inherent strengths and abilities of people, rather than the deficits or problems, acknowledges the wealth of knowledge and experience people with such conditions have, and builds relationships and partnerships between people and health care partners.

Potential limitations of this work need to be acknowledged. The case study approach may limit the generalizability of the findings to other regions and cultures in New Zealand, and internationally. To undertake value-based research of this type takes time and cannot be rushed—time to get to know the communities and develop mutual respect and trust—yet the sociopolitical landscape can rapidly change and render the findings less influential as political standpoints and polices transform.

We firmly believe that if health care is culturally appropriate and made accessible and equitable for our 3 targeted groups, applying the concept of universal design, it will enable all people in New Zealand to have improved health. Lessons learnt from our 3 case studies can thus be extrapolated to provide learning points for New Zealand health services with the aim of improving health at the community (primary care) level, which in turn may reduce the health care required at secondary and tertiary levels. We emphatically hold the view that people are the best experts in their own health conditions—they have the lived experiences within their own life contexts. However, for many people, especially the population groups we seek to work alongside, mainstream policies (pertaining to both health and beyond) and health and disability initiatives frequently leave them behind, and they simply become “the forgotten” [110].

## Appendix

Multimedia Appendix 1 Peer review report from the Health Research Council of New Zealand.

Multimedia Appendix 2 23-448-Hale 53 Applicant Peer Review.

Multimedia Appendix 3 23-448-Hale 81 Applicant Peer Review.

Multimedia Appendix 4 23-448-Hale 91 Applicant Peer Review.

## Abbreviations

DPO: disabled person’s organization
RE-AIM: reach, effectiveness, adoption, implementation, and maintenance
SDM: supported decision-making
TCC: The Cause Collective
