# Supplementary material for: Taunakitanga Takitini, Reframing Self-Management Support for All in Aotearoa New Zealand: Protocol for a Participatory Case Study Program of Research
Source: JMIR Res Protoc. 2026 Apr 8;15:e89658. doi: 10.2196/89658 (PMC13103644; doi:10.2196/89658)
Supplement: Multimedia Appendix 1 [file resprot_v15i1e89658_app1.pdf]

Applicant peer review report

Reviewer # 2

Proposal details

Title	Taunakitanga Takitini: reframing self-management support for all in Aotearoa
First named investigator	Professor Leigh Hale (University of Otago)

Rationale for research — Score: 5

The research topic is important and justifiable to Aotearoa. The underlying purpose of this research is to increase the health and wellbeing of those most vulnerable and in need, and the researchers should be commended for that. It is also timely; I find particularly convincing the argument that in times of rapid transformation, those most in disadvantage are more likely to miss out from opportunities to benefit from reforms. Addressing some presentation and methodological issues would help to improve the application. I have made below some comments to further strengthen the rationale.

The aims and research questions are appropriate, clearly presented and build on existing knowledge. I have made below some suggestions to further strengthen them. There is appropriate responsiveness to Māori. The approach is culturally responsive and appropriate, and aligned with the principles of Kaupapa Māori, contributing to ensuring the outcomes of the research will serve the intended objectives.

Key populations. I commend the focus on three key populations living with lifelong conditions and frequently underserved. While the benefit of focusing on Māori, Pasifika and those with learning disability is beyond doubt, the justification of this selection would be stronger if evidence is provided or more clearly presented around how these groups are underserved specifically regarding supported self-management (SSM) services/options, and not around heath in general.

Besides, one would like to know if there are other groups that are also particularly disadvantaged regarding SSM, and which the proposal are not considering. I may have missed it, but I see no mention of other population groups who may also be left out from services and having lower health outcomes. For example, in Aotearoa, Asian population have lower enrolment rates with a PHOs, suggesting weak connection with primary health system. Internationally, homeless and recent immigrant people are often disconnected from health services. What are or may be other disadvantaged groups in relation to SSM, and not considered in this proposal, if known? How will the research team facilitate that the emerging findings would also benefit other disadvantaged groups? I acknowledge that the focus is already wide in including three groups (Māori, Pasifika and those with learning disabilities). Likely, there would be not one but multiple emerging models of care that would work for different population characteristics.

Current model description in study justification. After reading the proposal, I am still unclear of the current model/s of (supported) self-management in Aotearoa. The proposal would improve by more clearly presenting what the current model/s looks like, in which areas it is deficient, and what is the evidence for this, so that the reader can understand where the frontier of knowledge lies. It seems to me a more precise/explicit description of the ´business as usual´ is relevant for a more solid justification of the study. How do we know that what is needed is new models of care instead of, for example, focusing on overcoming the barriers to participation from groups more in disadvantaged and indigenous groups (ref 20)? Is it the model that is wrong, or rather that its implementation has been deficient? Are refs 17-21 NZ or internat? Ref 24 is NZ or internat? Another example, what do we know about current SSM not being holistic enough in its approach? What is the evidence substantiating that criticism? I am not questioning the statements made, just pointing out that I am missing a more explicit description/assessment, and the sources for such information. This assessment would also help with the data collection phase.

International consideration. I may have missed this from the proposal, what specific examples are provided from other sectors or other countries on how the application of a more comprehensive SSM approach have led to positive impacts in the health and social sector, and particularly for beneficiaries? What have been the lessons learnt?

Inclusion of service providers. Communities´ inputs will inform on what they need from the services and what they are

getting or not from them. I wonder if the study would include service providers as well and how? It´s fantastic to focus on the users to include their perspectives, but if the aim is to make recommendations on what the services should and could look like and how that could be implemented, it seems to me the perspectives of providers are also important. I acknowledge this may be tricky as the focus is to maintain the leading role of beneficiaries.

Characterizing current model/s of care as another objective or RQ. The research seeks new or refined models of care, and for that we need to properly understand the current ones. I miss an intermediary objective between objective 1 and 2 for characterizing what the current models are, and how they aim to support whanau and communities. In my view, before developing the new models, we need to understand the existing models too, so as to identify where there needs to be changed, e.g.is it more in the design of existing models, or in the implementation? Is the problem that Māori, Pacific and people with disability are not accessing services, or that the services accessed do not respond to their needs sufficiently? It would require to also add another Research Question on characterizing what is the current model of care, to help understand where and to what extent it is deficient in relation to aspirations to living well of Māori, Pacific and people with learning disabilities.

Time- Programme research questions Re. RQ ´How beneficial are these refined or new models?` The 1-2 year time-frame seems too short to show full benefits of the new or refined models of care. Sustained benefits often require longer time. I suggest to acknowledge this as a limitation and to add thoughts on how to address it, e.g. what benefits may be more likely/expectred to show after study completion?

´Home grown´ as justification. I am not convinced by how the argument of ´homegrown´ is used to justify the study. ´Homegrown´ does not guarantee that something works. And the fact that an initiative is imported does not necessarily mean that it will fail when applied in Aotearoa. I would suggest authors to revise/refine these statements around whether initiative works or not. Rejecting what we have because it came imported from elsewhere does not seem a good enough reason. The justification lies in to what extent and how it is not working and for whom more than on the origin. Homegrown initiatives are more likely to work as they take into account the local context.

Design and methods Score: 6

The study seems well designed to achieve the multiple aims. A key strength of the proposal is the inclusion of Māori, Pasifika and those with learning disability. I want to acknowledge the extra effort needed, and often under-valued, when including multiple and distinctive population groups as in this case.

Kaupapa Māori. The proposal shows excellent culturally appropriate methodology and responsiveness to Māori, not as a side issue, but central in the design of the study, including the study team composition Prioritizing rangatiratanga to enable Māori to make decisions about research and how services could be framed to benefit them is central in advancing Aotearoa´s aspirations for health. This will also facilitate the management of patient safety issues, as acknowledged in the application. I want to commend as well the acknowledgement of Te Mana Raraunga, Māori data sovereignty guidelines. In addition, the capability approach is a key strength in matching research aims and methods. I find another strength of the methods is the wide range of outcome assessment indexes, including international and group-specific ones.

Current models of care. As noted in the earlier section, I find the methods need to consider including the description/assessment of current models of care, to help with the identification of gaps and to reframe them to better serve the aspirations and needs of users and communities. Related to this, I see in p2: 11, `Interviews will also focus on how disability support and health care professionals engage and or support them to understand and manage their own health`. This is a first step but it may not be sufficient. Providers´ insights would also be useful in informing what parts of the current models need to change to cover the identified gaps. To make best use of information compiled from end-users, I would suggest adding another RQ on assessment of current models of care.

Regarding Pacific group, the proposal states that they will establish connections with expert reference group, community leaders and key stakeholders. Pls clarify the extent of such connections so far. They seem to be well established for the other two groups and the way it is narrated for Pasifika group it gives the impression of being less established? but it may be only a matter of the wording. Besides, for new connections, Zoom may not be acceptable, particularly for Pasifika communities and more in-person meetings would be expected.

Participant selection. Could more details be provided on participant selection and recruitment. For example, how will participants be selected? Would the participants for Obj 3 be the same ones as in Obj 1 and 2? To what extent would researchers consider how the communities selected are representative of the wider target group? Also, what could be the unintended consequences for participants? Given the instrumental role of the associations in selecting study participants, could there be some potential conflict anticipated, and if so, how to mitigate risks? These considerations seem important to ensure the integrity of community voices will be maintained.

Milestones: For Year 1- data gathering exercises completed- maybe refine of data gathering for first year completed, as we may expect data gathering continuing throughout?

Timing- In general, feasible, achievable within the time frame. Year 1 to set up the team and conduct data collection for Obj 1 and 2 seems optimistic and may be worth to account for possible delays.

Principles – It may be worth if sufficient space, to add briefly on the Whanua Tuatahi Research principles developed by Jones

Funding- Is the separate founding for PhD or Master candidates secured already?

Longitudinal surveys- how many and when?

Research impact **Score: 5**

The research has the potential to directly inform change in policy and practices in the health and disability sector. First, because of working in close partnership with stakeholders, from communities to providers. Second,using and valuing Māori and Tongan knowledge and wisdoms. The pathway for research impact is provided. Yet, I suggest strengthening the Research Impact section in the Summary, which does not seem to properly reflect the strengths shown in the application; providing more specifics would help.

Impact on wider population groups. The explicit target of health equity is a strength in the application, and it could be improved by reflecting how findings may impact on other groups, including other groups in disadvantaged.

Prioritization. As impact is sought at multiple levels (e.g. whanau, as well as research agenda, services,policymakers) it may be worth to reflect on prioritization as well as how the impact across those level may act in a synergic way.

Partners excellence. The project is in collaboration with best practices such as Turanga Health, THS and the DBI / People First are exemplars of Aotearoa. The partners selected seem to be of recognized excellency in providing healthcare and social services, proactive, etc., and this a key strength of the study. On the other hand, this focus on top partners is likely to influence the models that will emerge, and may condition that those models may work for well-functioning providers, but maybe not so for other partners in not such a strong position? How do authors plan to address this ´selection bias´ to ensure emerging findings are applicable to a wider range of providers, to maximize impact?

Risk recognition. Module2: 17: “our findings will be incorporated into Aotearoa healthcare decisions” Maybe add ´potentially´ or similar, as it is an objective but cannot be guaranteed. What if Te Aka Whai Ora will not embrace the new or refined models developed for example? In times of transitions and reforms, the direction of health organizations or priorities may change unexpectedly.

Potential for outcomes Score: 5

The proposed research has strong potential for positive health impact and outcomes, particularly among population groups traditionally underserved. It will provide very important knowledge from patient´s perspective. It also has a training component, in particular for young investigators and targeting when possible Māori and Pacifica and people with disabilities. Although there is a reference in the proposal to economic considerations, it doesn´t seem to be further considered in the outcomes section, as it is not an objective of the research. It will also contribute to enhance understanding to the nature and meaning of ´support´ and ´care´.

Extrapolation to other groups/contexts. I think the potential outcomes section could be strengthening by reflecting on how will findings and knowledge and lessons produced be extrapolated to other similar groups in Aotearoa, as well as to different population profile groups, and service provider groups. What specifically will be done to facilitate the transfer of knowledge to different contexts, if it is an objective of the proposal? It would be helpful to add further thoughts on how to increase the transferability of results to other groups and settings, including regarding providers who may not be such a track success record.

Expertise and track record Score: 6

Impressive team pulling Māori and Pasifika and wide range of stakeholders. The expertise, track record of the research team and involvement with Māori, Pasifika and people with disabilities communities is a key strength. In addition, the research team has excellent connections to policy and with providers, as demonstrated in the application. If anything, maybe the expertise on SSM in the team may be highlighted further (is probably there but it doesn´t come up as strongly as the community and policy links).I particularly appreciate the experience in dissemination of results via a diverse range of initiatives. Cultural appropriateness seems guaranteed by the team composition and approach, clearly stated

Collaboration and integration Score: 5

All aspects of collaboration and integration seem well covered by the research team.

Knowledge sharing between the three groups. I find the application could improve by providing more details on how the the research across the three groups (Māori, Pasifika and people with disabilities) will exchange insights and findings and serve to build on each other. Would there be comparisons made across the three groups? How will the emerging lessons from the three studies be put together?

General comments

What stands out in this proposal for me is the capabilities approach, to work with the community, to construct together aspects of the research activities, the assumption being that people are the best experts in their own health conditions. The research will favor bottom-up rather than top-down approaches, thus boosting the potential to have direct meaningful impact and resonance for those it is aiming to serve.

I have not been able to review the budget due to time constraints.

I look forward to seeing the results. It will offer key insights for aspirations for our health and disability system that would help directing and keeping accountable ongoing transformations.
