# Supplementary material for: Taunakitanga Takitini, Reframing Self-Management Support for All in Aotearoa New Zealand: Protocol for a Participatory Case Study Program of Research
Source: JMIR Res Protoc. 2026 Apr 8;15:e89658. doi: 10.2196/89658 (PMC13103644; doi:10.2196/89658)
Supplement: Multimedia Appendix 2 [file resprot_v15i1e89658_app2.pdf]

Applicant peer review report

Reviewer # 53

Health Research Council of New Zealand
Te Kaunihera Rangahau Hauora o Aotearoa

Proposal details

Title	Taunakitanga Takitini: reframing self-management support for all in Aotearoa
First named investigator	Professor Leigh Hale (University of Otago)

Rationale for research Score: 6

The application speaks directly to how the programme of research not only aligns but propels health sector strategies amid current national, regional and community health reforms, challenges and solutions.The research team have thought carefully about how supported self-management must be reframed to fit our context here in Aotearoa, and the aims and research questions are focused on developing this understanding across three discrete research projects and communities.This includes a Māori focused project in Te Tairāwhiti, and one within the Tongan community in Tāmaki Makaurau.The application could have also referred to Whānau Ora - a kaupapa that certainly speaks to the rangatiratanga of whānau in decision making, and acknowledges that when well supported, whānau hold solutions to any challenges that they may face.International citations for SSM are provided, though I would encourage the applicants to think about the relevance of and learnings from this research programme, particularly for other Indigenous communities internationally.

Design and methods Score: 6

The three discrete research projects within this programme are founded on relevant methodologies, and will utilise methods appropriate for each of the contexts within which the projects will be conducted; objectives proposed can certainly be achieved within the timeframes given.What is revolutionary is that the research programme begins with developing an understanding of the aspirations and challenges for the targeted populations and those servicing them.This will be a real strength of the research programme, and will certainly be of interest to other researchers who may wish to emulate a similar research process or focus in the future.Often, community based services and clients are focused on the day-to-day challenges of service delivery and living well; some thinking, therefore, is required on how the research intends to lift this focus, and how the research will encourage discussions around aspirations.

Research impact Score: 7

The impact of this research is extremely exciting, as it intends to address equity within the health system.The way in which the research programme has been designed ensures that the research is led by the communities within which the three discrete projects are based, and will address issues identified by these communities.The development of healthcare models that seek to service and lift the health and wellness of these communities (consisting of disadvantaged populations) is paramount, so too is the emphasis on enduring relationships with community, service providers, and policy makers, to ensure that the findings from this research are not only disseminated, but that there is uptake of recommendations stemming from it.The research team certainly have the connections, experience and attributes to deliver.

Potential for outcomes **Score: 7**

As previously stated, the proposed research certainly addresses inequities in the health sector, and there is a clear intent to influence health policy and service provision that will impact on disadvantaged populations, their whānau and communities.The research programme will certainly provide training opportunities through the provision of research interns based within the three discrete communities (therefore, Aotearoa focused), but also professional development opportunities for those who already service these communities.

Expertise and track record **Score: 7**

The research team are certainly qualified and experienced to lead out on this programme, and it is good to see that resource has been dedicated to each of the three sites, including community-based FTEs and summer/winter interns.

Collaboration and integration **Score: 7**

As previously stated, research team members are well qualified, and have the experience and knowledge to undertake this work.A real strength of this application is the involvement of community-based organisations in leading the three discrete, community-based projects.FTEs across the programme and project personnel appear to be appropriate, and the proposed research management protocols (e.g. regular meetings) will ensure that research skills are synergised, and that the programme is managed well.

General comments
