# Supplementary material for: Taunakitanga Takitini, Reframing Self-Management Support for All in Aotearoa New Zealand: Protocol for a Participatory Case Study Program of Research
Source: JMIR Res Protoc. 2026 Apr 8;15:e89658. doi: 10.2196/89658 (PMC13103644; doi:10.2196/89658)
Supplement: Multimedia Appendix 3 [file resprot_v15i1e89658_app3.pdf]

Applicant peer review report

Health Research Council of New Zealand
Te Kaunihera Rangahau Hauora o Aotearoa

Reviewer # 81

Proposal details

Title	Taunakitanga Takitini: reframing self-management support for all in Aotearoa
First named investigator	Professor Leigh Hale (University of Otago)

Rationale for research — Score: 6

This is an innovative research, incorporating Maori, Pacifika and mainstream disability and addressing the inequities they experience.

The knowledge I have of these researchers is that all of them have years of experience working and re sea Chiang the disability community in their fields, they have a good diverse demographic and the research itself appears to be robust in a way that will achieve the outcome they seek

Design and methods — Score: 6

By having several disabled researchers and incorporating te tiriti constructs along with the other cultural identities, this will address disability from a global south construct and not the dominant bio Western frameworks that often dominate disability discourse

Research impact — Score: 7

Apart from the dominance of global north westernised concepts of disability, this is an opportunity to finally provide the Polynesian or global south perspectives on disability which will definitely benefit Aotearoa/New Zealand and increase the knowledge, thereby closing the gap in knowledge that has existed in health and disability policy

Potential for outcomes — Score: 6

There is no doubt with these researchers, there is an opportunity to ensure the knowledge will provide valuable info for providers etc, the fact they are working with te whatu ora with the two CEO's who are embracing diversity in health, this will potentially bring outcomes the Health report leading to the new ministries offering a way of being more inclusive of a very marginalised group

Expertise and track record — Score: 7

most of these researchers have extensive backgrounds in disability and at least two of them have lived experience. They have set up a team with extensive knowledge

Collaboration and integration Score: 7

Yes, it shows a very good team for collaborations and collation of information etc

General comments

As a disabled person with a background in indigenous research, I have seldom found research in this area and now it's clear, this experience and knowledge is growing. I strongly encourage research of this type
