# Supplementary material for: Taunakitanga Takitini, Reframing Self-Management Support for All in Aotearoa New Zealand: Protocol for a Participatory Case Study Program of Research
Source: JMIR Res Protoc. 2026 Apr 8;15:e89658. doi: 10.2196/89658 (PMC13103644; doi:10.2196/89658)
Supplement: Multimedia Appendix 4 [file resprot_v15i1e89658_app4.pdf]

**Applicant**	Professor Leigh Hale	**Respond by**	14/03/2023
**Assessing Committee**		**HRC Reference**	23/448
**Title of Research**	Taunakitanga Takitini: reframing self-management support for all in Aotearoa		

Rationale: We thank R#2 and R#81 for their compliments.

Here we address R#2 and R#91's queries about current models of SSM in NZ (local versus imported),the research gap, and a current models of SSM research question: The NZ Ministry of Health (MOH) have supported development of resources and tools for primary care teams to enable people with long term conditions to be supported to self-manage daily life (i.e. "self-management support"), with a link to the Health Navigator website (Te Whatu Ora website, 2023). On the latter website 2 (of 3) self-management programmes listed are international (3rd is paediatric), Stanford Model and Flinders Program, citing use of these by many NZ organisations. Flinders Program focusses on training healthcare professionals to work in a structured way with individual emphasising concordance (e.g. designing a Care Plan identifying main problems; agreed goals; agreed interventions; sign off by both patient and health professional with review dates). Stanford Model are community-based sessions (held once a week for 6 weeks) educating individuals to manage their health condition.

Both programmes emphasise the "self" (personal responsibility and an expectation to manage themselves), a concept that is a cultural artifact, a way of thinking that comes from white, Western, neoliberal countries.[1-3] Culture shapes how a person makes sense of themselves as the "patient" and as the "health professional," and thus central to how a person lives with an "incurable" health condition.[1-3] Our research (p10) challenges these notions, aiming to reframe "supported 'self'-management" as opposed to "'self'-management support" (SMS) for the Aotearoa context in which many Māori and Pacific people privilege collectivism over the self. The current SMS MOH-endorsed approaches have mixed outcomes, working better for some sub-groups.[4] For example, exercise and education interventions promoting SMS are attended by those already self-managing their condition (hence their attendance).[5] Technology found to assist SMS remains condition-specific, e.g. persistent pain.[6] Culturally and linguistically diverse people living with multi-morbidity and in low socioeconomic conditions report being motivated to SM, but have challenges understanding their conditions and how to manage them, influenced by their health beliefs and health literacy.[7] For people living with severe multimorbidity, introducing SM strategies have however resulted in: "*a cohort of tired, distracted patients struggling to manage their lives in the face of multiple conflicting challenges, with insufficient energy for the level of personal agency required to deal with the self-management approach*".[8] Research entitled "*How does it feel to be a problem?*" concluded that little attention has been given to the "how" of SM in Aotearoa.[9] Whilst many SM techniques are described (e.g. goal setting), the active "ingredients" of SM are still largely un-evidenced, likely due to its contextual nature.[10] A capabilities approach to collaborative, supportive and enabling relationships is one in which collectivism-based communities (such as Māori and Pacific communities) already exemplify and are thus communities potentially well-placed to advance knowledge of the "how" of SSM. Research specifically contextualising SM for Aotearoa is growing but is still largely condition specific: *Taking Charge Session* (post-stroke),[11] *Mana Tū* (diabetes),[12] *Living Well Toolkit* (neurological),[13] *Care Plus* (high health needs).[14] Counties Manukau Health initiation of Manaaki Hauora (Supporting Wellness) campaign resulted in a guide to culturally appropriate SSM condition-based interventions.[4] Supported SM is possible even for those deemed to have limited autonomy, such as those experiencing learning disabilities, often challenged by attitudes, needs and personal values posed to their decision making.[15]

Our innovative research, addresses this knowledge gap, challenging the status quo and current notions of "'self'-management support" (i.e. providing resources and tools) and seeking to reframe "supported 'self'-management" (i.e. how can we support whānau) timeously as the new health reforms foundationally embed Te Tiriti, equity and a population health approach (Pae Ora legislation).

R#53: We were remiss in not referring to Whānau Ora in our application. This approach to wellbeing that concentrates on whānau as the decision-makers who determine their goals and aspirations, is arguably an existing model of care that meets our aspirations; however, it has a limited reach in NZ and is more widely and socially focussed than our target population of those living with lifelong health conditions or disabilities. We will certainly draw on Whānau Ora going forwards. As SSM research in indigenous populations worldwide is limited, programmes tend to be based on those developed in westernised paradigms with

inadequate evaluation,[16,17] our programme will potentially generate Aotearoa specific knowledge and build indigenous knowledge internationally.

R#2: We acknowledge queries (i) We would like to extend to Asian and refugee populations, but there are time and resource limitations. Our premise of challenging the "self" in SM programmes is however likely to hold true for many of such groups who value collectivism over the individual. (ii) In this research we are actively partnering with two service providers (Turanga Health and Tongan Health Society) in which staff and managers will be actively involved as participants. (iii) We recognise the limitation of the short evaluation time frame available in this programme, providing an opportunity for future research endeavours.

Design and methods: R#2, R#53, R#81: Thank you for your compliments. We agree that we must challenge and encourage discussions around aspirations rather than focus on the everyday in our data gathering activities. And this, (R#2) may take longer than the currently allocated milestone of year 1.

R#2: (i) We apologise for the confusion regarding the Tongan Health Society. Their expert reference group, community leaders and key stakeholders are already well established and consulted frequently on the Society's business. This study was written by the CEO/Medical Director (NI Doherty) and Research Director of this Society (co-Director Dewes), and they consider zoom appropriate if necessary (mitigating risk during Covid times). We fully acknowledge that culturally face-to-face is preferable. (ii) In keeping with our methodology, we intentionally have not pre-determined participant selection and recruitment for each study. These will be established later in partnership with our community partners and their communities and aim to capture diverse perspectives. But R#2's comment regarding conflict of interest and maintaining the integrity of the community voice are well noted and will be considered. (iii) Briefly, the Whānau Tuatahi Research principles are: whakawhirinaki (trust), whakawhanaungatanga (relationship building), whakamana (empowerment), ngāwari (flexibility), utu (reciprocity) and hurihuringa (reflexivity) (p12). (iv) For funding of PhD and Masters we intend to leverage off other existing resources to enable the programme budget to appropriately cover the research costs. Our team has a successful track record of obtaining graduate research funding for Māori and Pacific candidates via the HRC, University of Otago and the Ministry of Pacific Peoples Toloa scholarships (p22, #4). (v) As stated (p14), we will undertake qualitative interviews and likely two qualitative surveys (determined in Objectives 1 and 2) in our Qualitative Longitudinal Research every 3 months for 2 years.

R#91: (i) We were pleased for the appreciation of the importance to not perpetuate epistemic injustices inherent in existing scholarship. Thus, our epistemological stance (p12) meant we intentionally developed our research questions to be open and broad, creating space for refinement, through our methodologies, by our community partners. This stance enables the research (both questions and measurement) to be created and accepted by communities (thus prioritising rangatiratanga) rather than through post- positivist epistemic logic. (ii) We agree that whilst our three studies are distinct there is definite intersectionality (as all three community partners will indeed work with Māori, Pacific, and disabled participants). This is described on p13 via the conceptualisation of our research (study metaphor, illustrated in Figure 1). We will exchange insights and findings from our three studies via our annual in-person hui (p21) and governance group and study leads huis (p22). For example, in study 3 we will focus on the role of SDM in SSM, but as pointed out by R#91, learnings from this study will indeed be applicable and valuable to all. (iii) Regarding an evaluation framework, we will be using the RE-AIM (reach, effectiveness, adoption, implementation, and maintenance) model (p13). (iv) This programme of research has at its core collaboration, partnership and relationship building, and thus objectives 1 and 2 are foundational and all-important to its methodologies, and as expressed by R#2, may take longer than the 1-year milestone. These collaborations started long before the application (as evidenced by the community partners developing their own studies and as described in our Research Kaupapa, p11) but need to be undertaken more formally as part of this programme of research to strengthen their importance.

Research Impact: We thank R#2, R#53 and R#81 for their compliments. R#2: As equity was considered strong, we believe our findings will have impact on other disadvantaged groups with its strengths-based

approach and consideration of collectivism communities, which will hold true for many Asian and refugee groups. We agree there is selection bias, we purposely chose to work with "well-functioning" providers as a strengths-based approach, to evaluate and champion exemplar models that will be attractive for possible Te Aka Whai Ora commissioning and for our findings to potentially be incorporated into Aotearoa healthcare decisions. R#91: We will evaluate the new models improve service delivery and outcomes using the RE AIM model. We acknowledge that we do not provide details regarding impact, but this is in keeping with our research Kaupapa; to uphold rangatiratanga as our community partners have sovereignty, autonomy, control, and independence over their data. P14-17 provide explanations as to why our community partners are considered exemplars in their respective areas.

Potential for outcomes: We thank R#2, R#53 and R#81 for their compliments. R#91: We acknowledge that our research Kaupapa prevents a full description of communicating our findings and evaluating impact; these will be guided by our community partners as the research progresses.

Expertise and track record: We were encouraged that all four reviewers considered our research team to be a strength of this proposal. To highlight team members with SSM expertise, please refer to the publications of Hale, Wilkinson, Perry, McKinlay and Trip. Our international advisory board members with SSM expertise are Jones, Oosman, Lawn and Watkin.

Collaboration and integration: We thank R#53 and R#81 for their compliments. R#2: We will exchange insights and findings from our three studies via our annual in-person hui (p21) and governance group and study leads hui (p22). Whilst they are three distinct studies there is definite intersectionality. This conceptualisation is described on p13 (study metaphor, Figure 1).

General: We thank R#2 and R#81 for their compliments. R#91: Our overall research programme is underpinned by Whānau Tuatahi Research (Māori) and Fonua (Tongan) conceptual frameworks, prioritising rangatiratanga (self-determination). The REAIM model will guide implementation evaluation. Our studies are integrated by their focus on people living with lifelong health conditions or disabilities.

REFERENCES: 1. Hale L, Oosman S, Stewart AV. (2022) Editorial: Challenging the concept of self-management support in unique and diverse populations. Front. Rehabilit. Sci. Sec. doi: 10.3389/fresc.2022.999528. **2.** Wilson L, Wilkinson A, Tikao K. (2022) Health professional perspectives on translation of cultural safety concepts into practice: A scoping study. *Front. Rehabilit. Sci.* 3:891571. doi: 10.3389/fresc.2022.891571. **3.** Basurrah AA, Al-Haj Baddar M, Di Blasi Z. (2022) Positive psychology interventions as an opportunity in Arab countries to promoting well-being. Front. Psychol. 12:793608. doi: 10.3389/fpsyg.2021.793608. **4.** Framework Trust. Enabling self-management support. **5.** Hale L, Higgs C, et al. (2022) Evaluating the effectiveness of an equity focussed Diabetes Community Exercise Programme for management of diabetes. eClinicalMedicine, 46: 101361. **6.** Devan H, Hale L, et al. (2018) What works and does not work in a self-management intervention for people with chronic pain? Phys Ther Reviews 98(5):381-397. **7.** McKinlay E, Graham S, Horrill P. (2015) Culturally and linguistically diverse patients' views of multimorbidity and general practice care. J Prim Health Care 7(3):228-235. **8.** Francis H, Carryer J, et al. (2018) Self-management support? Listening to people with complex co-morbidities. Chronic Illness. 0(0):1-12. **9.** Sheridan N, Kenealy T, et al. (2019) How does it feel to be a problem? Patients' experiences of self-management support in New Zealand and Canada. Health Expect 22(1):34-45. **10.** Dineen-Griffin S, Garcia-Cardenas V, et al. (2019) Helping patients help themselves: systematic review of self-management support strategies in primary health care practice. PLoS ONE 14(8):e0220116. **11.** Harwood M, Weatherall M, et al. (2011) Taking charge after stroke: promoting self-directed rehabilitation to improve quality of life. Clin Rehabil. 26(6):493-501. **12.** Harwood M, Tane T, et al. (2018) Mana Tu: a whānau ora approach to type 2 diabetes. NZMJ 131(1485):76-83. **13.** Sezier A, Mudge S, et al. (2018) Development of a toolkit to enhance care processes for people with a long-term neurological condition. BMJ Open 8(6):e022038. **14.** Askerud A, Jaye C. (2020) What is the answer to the challenge of multimorbidity in New Zealand? J Prim Health Care12(2):118–121. **15.** Hale LA, Trip HT, et al. (2011) Self-Management abilities of diabetes in people with an intellectual disability living in New Zealand. J Policy Pract Intell Disabil 8(4):223-230. **16.** Moore E, Lawn S, et al. (2019) Self-management programs for Aboriginal and Torres Strait Islander Peoples with chronic conditions: A rapid review. *Chronic Illness*15(2):83-123. **17.** Kulhawy-Wibe S, King-Shier KM, et al. (2018) Exploring structural barriers to diabetes self-management in Alberta First Nations communities. *Diabetol Metab Syndr* 10, 87.
